# Spatial analysis of global Bitcoin mining

**DOI:** 10.1038/s41598-022-14987-0

**Published:** 2022-06-23

**Authors:** Wei Sun, Haitao Jin, Fengjun Jin, Lingming Kong, Yihao Peng, Zhengjun Dai

**Affiliations:** 1 Key Laboratory of Regional Sustainable Development Modeling, Institute of Geographic Sciences and Natural Resources Research, Chinese Academy of Sciences, Beijing, China; 2 College of Resources and Environment, University of Chinese Academy of Sciences, Beijing, China; 3 School of Computer, Beijing Information Science and Technology University, Beijing, China; 4 BTC Team, BIT Mining Limited, Hong Kong, China

**Keywords:** Environmental social sciences, Environmental impact, Sustainability

## Abstract

Bitcoin mining is not only the fundamental process to maintain Bitcoin network, but also the key linkage between the virtual cryptocurrency and the physical world. A variety of issues associated with it have been raised, such as network security, cryptoasset management and sustainability impacts. Investigating Bitcoin mining from a spatial perspective will provide new angles and empirical evidence with respect to extant literature. Here we explore the spatial distribution of Bitcoin mining through bottom-up tracking and geospatial statistics. We find that mining activity has been detected at more than 6000 geographical units across 139 countries and regions, which is in line with the distributed design of Bitcoin network. However, in terms of computing power, it has demonstrated a strong tendency of spatial concentration and association with energy production locations. We also discover that the spatial distribution of Bitcoin mining is dynamic, which fluctuates with diverse patterns, according to economic and regulatory changes.

## Introduction

The validation of Bitcoin transactions is enabled by its proof-of-work (PoW) consensus mechanism^1^. Bitcoin miners perform scanning for hash value to compete for obtaining the right of recording the block of transactions, and the successful creator of each block is rewarded by a certain amount of bitcoins. This process is called ‘Bitcoin mining’^2,3^. At the very beginning, mining activity was only supported by a few participants equipped with regular computers^4^. The surge of Bitcoin price and mining profitability incentivized increasing computing power to participate in the game. Moreover, specific mining rigs were quickly designed, manufactured and upgraded^5^. Mining sites were purposefully selected and developed. Huge amounts of energy and resources were put into mining industry^6–8^.

Bitcoin and its mining activity have aroused attention in a variety of fields, including but not limited to blockchain technology^2,3^, financial econometrics^9,10^, and sustainability issues^7,8,11–14^. Exploring the spatial distribution of Bitcoin mining will provide new angles and evidence with respect to a large portion of extant literature. In particular, the investigation from a spatial perspective will help to verify the decentralized design of blockchain technology, to identify certain kinds of price effects on cryptocurrencies and to make accurate estimations on energy consumption and carbon emissions from mining activity.

Some sustainability studies have brought valuable tracking ideas and provided interesting mapping outputs into spatial aspect of mining activity^15–18^. Nevertheless, the spatial analyses as by-products from these studies are still limited in terms of data granularity and analytic methods. On the other hand, geographers and economists have a long tradition to describe geographical locations, patterns and dynamics of human production and trading activities^19–22^. Bitcoin mining behaves quite differently in space when compared to conventional industrial activities. However, there is barely any novel idea published with regard to this nascent activity. Therefore, in this paper we aim to fill this gap by investigating the spatial patterns, characteristics and shaping forces of mining activity, as well as to understand, from a spatial perspective, the implications to the aforementioned topics from adjacent fields.

We carried out the research by extracting the hash rate data from million-level mining records and then desensitizing, geocoding and aggregating the data by hash rate, month and location (with unique longitude and latitude coordinates). To facilitate the spatial analysis, we divided the surface of the earth into hexagonal grids (n = 7205) and accommodated the hash rate data and the global power plant data^23^ within the same grid system through multilayer spatial join. We then explored the statistical analysis of spatial measures over the processed data sets. We disclosed four kinds of spatial phenomena of mining activity: diffusion, concentration, association and fluctuation. Furthermore, we put the results in the context of the drivers and stages of Bitcoin mining to better understand the causes for such spatial formations. The data sources and the step-by-step approaches are also detailed in the “Methods”.

### Basics of mining activity

Prior to diving into spatial analysis, we explain some basics of mining activity up front. Three key factors that influence Bitcoin miners’ behaviour are economic incentives, technological progress and regulatory schemes. Although there are a number of studies on the economics of Bitcoin mining^24–26^, we simplify the economic concepts of mining to better understand its relation with spatial choices as follows. In Eq. (1), *P*_*ij*_ is the mining profit for period *i* at location *j*, which is an important indicator for potential participants to determine whether they should enter the industry at the specific period and location. In Eq. (2), *GM*_*ij*_ is the gross margin for period *i* at location *j*, which is another indicator for miners to determine whether the mining rigs should be on or off.

1 $$ P_{ij} = TR_{ij} {-}FC_{ij} {-}VCA_{ij} {-}VCB_{ij} $$

2 $$ GM_{ij} = TR_{ij} {-}VCA_{ij} {-}VCB_{ij} $$

where *TR*_*ij*_ is the total mining revenue for period *i* at location *j*, which is determined by miner’s hash rate contribution, Bitcoins gained in the total network and exchange rate. *FC*_*ij*_ is the fixed cost for period *i* at location *j*, which consists of the amortization cost of hardware and initial settlement. *VCA*_*ij*_ is the variable cost (*Type A*) for period *i* at location *j*, which changes along with hash rate, mainly including the electricity cost. *VCB*_*ij*_ is the variable cost (*Type B*) for period *i* at location *j*, which also varies, but not strictly with hash rate, e.g., labour, bandwidth, cooling and other maintenance costs.

Three key takeaways are worth noting here: (i) any economic decision made by miners is based on the dynamics at a specific period and location but not on the static assumptions regardless of spatiotemporal factors; (ii) revenue factors are almost the same worldwide, while cost factors are highly localized. This means that miners obtain the same economic incentive regardless of where they are located. However, the cost breakdown of mining activity differs from location to location; (iii) it is difficult to achieve a real break-even point because of the high volatility of the Bitcoin price and the constant change in mining competition. 

Technological progress intensifies the arm race of mining activity and makes it ‘portable’. Mining hardware has quickly upgraded from central processing units (CPUs), graphic processing units (GPUs) and field programmable gate arrays (FPGAs) to application-specific integrated circuits (ASICs), with an exponential increase in computational performance and energy efficiency^5^. This has apparently influenced the aforementioned economic equations on both the revenue and cost sides. Meanwhile, a set of modern technologies (including communication, engineering, logistics, etc.) make mining activity able to move and relocate easily in space, as a ‘portable industry’.

Regulatory attitudes towards Bitcoin mining vary significantly jurisdiction by jurisdiction^27^. Some regulators take it favourable as data centre, cloud computing or fintech, while others treat it as a traditional energy-intensive industry or speculative bubble. Even within the same country, different sub-regions may hold totally different views. For example, mining activity was temporally banned in Plattsburgh, New York^28^, while it became more favourable in Austin, Texas, due to cheap electricity and a relaxed regulatory environment^29^. The lack of a clear global-level regulatory framework on how to define and regulate mining activity leaves room for Bitcoin miners to maneuver around the world.

Theoretically, mining activity is therefore free to move wherever it wants to exist. This is different from most industrial activities today, which are tightly constrained in space by two or more factors (e.g., resources, raw materials, talent and labour, market, transportation, regulatory permission). In addition, Bitcoin mining, to some extent, can be viewed as a prototype of the autonomous economy^30^ (Supplementary Note 2). That is to say, the algorithm, the economic formula and the built-in technology determine the suitable locations for mining and drive human activity to move accordingly.

### Spatial diffusion and concentration

**Figure 1:**
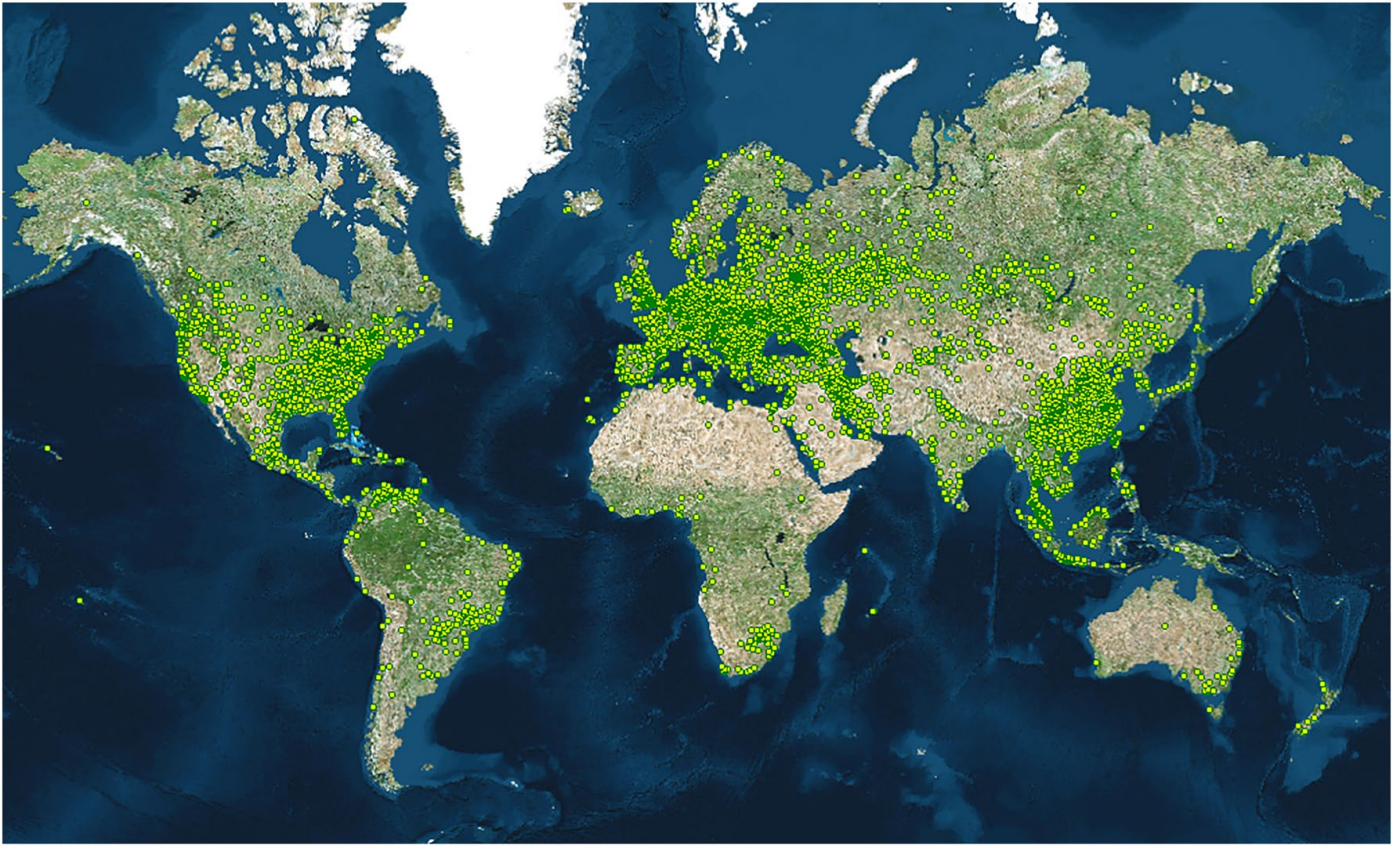
Global presence of Bitcoin mining activity. All mining locations detected (n = 6062) are mapped by their unique longitude and latitude coordinates. Details of each location are provided in Supplementary Table S2. The results are based on the monthly data from June 2018 to May 2019. The map is created by Geoda 1.18 (http://geodacenter.github.io/download.html).

**Figure 2:**
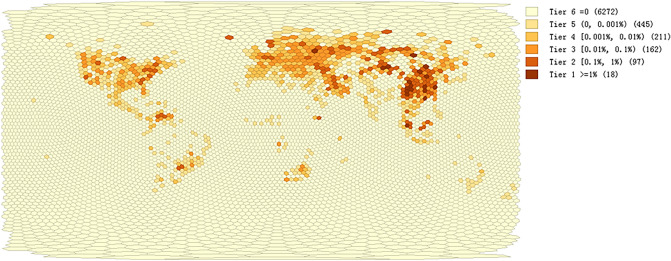
Share of computing power in terms of hash rate by grid. The share of computing power in each grid is represented as a percentage of total hash rates. All grids (n = 7205) are divided into six tiers with Tier 1 grids (n = 18, share of hash rate ≥ 1%), Tier 2 grids (n = 97, 1% > share of hash rate ≥ 0.1%), Tier 3 grids (n = 162, 0.1% > share of hash rate ≥ 0.01%), Tier 4 grids (n = 211, 0.01% > share of hash rate ≥ 0.001%), Tier 5 grids (n = 445, 0.001% > share of hash rate > 0) and Tier 6 grids (n = 6272, share of hash rate = 0). The results are based on the monthly data from June 2018 to May 2019. Details of the statistics are supplied in “Methods” and the repository as noted. The map is created by Geoda 1.18 (http://geodacenter.github.io/download.html).

It is natural to think that mining activity should be diffused all over the world due to its technical enablers and economic incentives. However, it is still astonishing to see how widely mining activity is distributed. By tracking the nodes connecting to one of the leading mining pools (“Methods”), we detected that mining activity existed in over 6000 geographical units from 139 countries and regions (Fig. 1). Except for well-known locations (e.g., China, Iceland, the US), mining activity was also detected at unexpected locations, such as Tahiti (the island in French Polynesia, the South Pacific archipelago) or Malawi (the landlocked country in Southeast Africa). If we divide the surface of the Earth into hexagonal grids (n = 7205), we notice that 933 grids, namely, 44.3% of Earth’s land surface (Supplementary Note 3), have been found to have Bitcoin mining footprint (Fig. 2). Owing to the arm race of computing efficiency, nonspecific machines were squeezed out, such as desktops, laptops, consoles and smartphones. Otherwise, it will be overwhelming in terms of spatial presence if all the spare capacities of those devices are put into mining activity.

**Figure 3:**
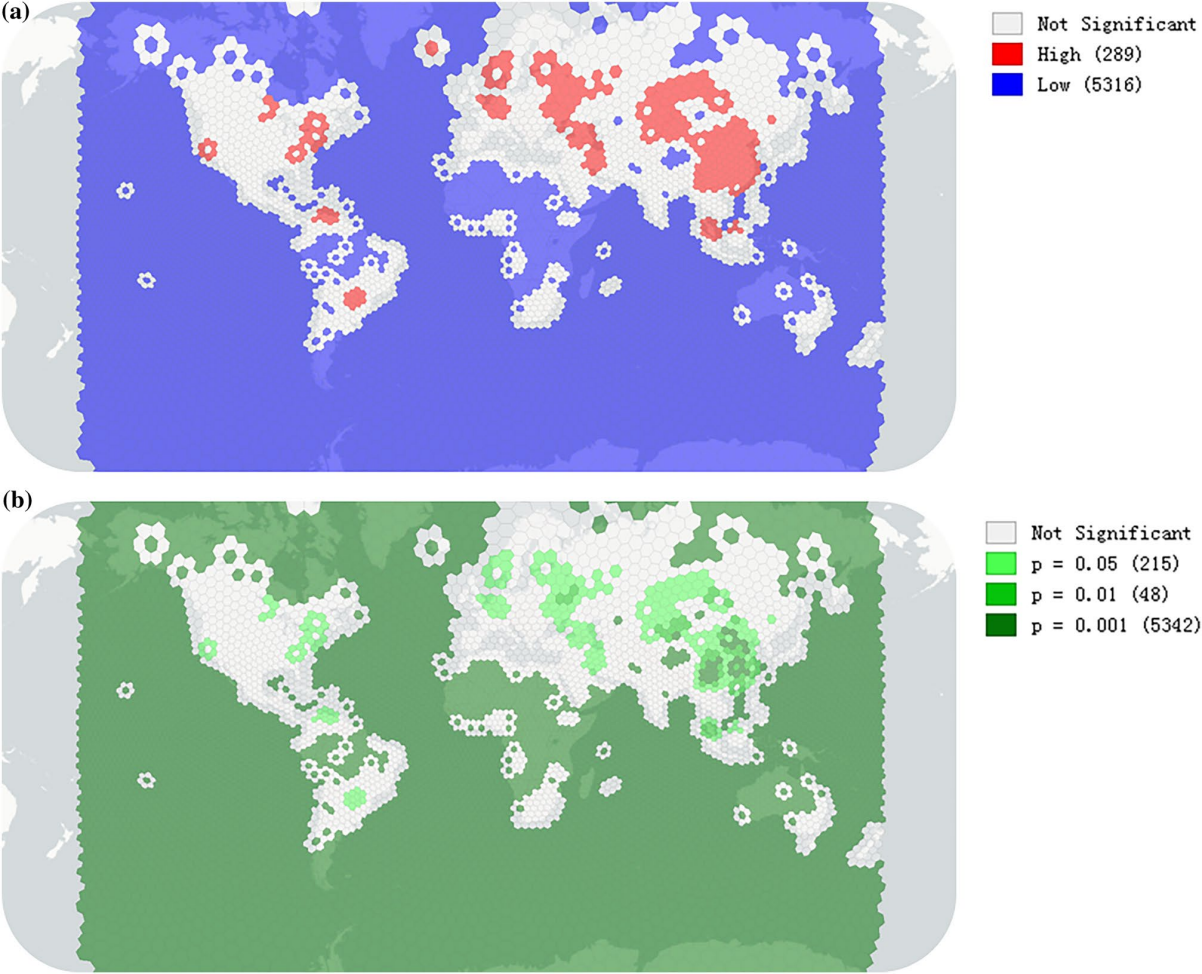
Hot and cold spots of Bitcoin mining activity with the corresponding significance map. (**a**) The hot spots (High-High clusters) and cold spots (Low-Low clusters) under the default setting of 999 permutations and a p-value ≤ 0.05 are marked in red and blue, respectively. (**b**) The corresponding significance map shows the clusters with the degree of significance reflected in increasingly darker shades of green, starting with 0.01 < p ≤ 0.05 (n = 215), then 0.001 < p ≤ 0.01 (n = 48) and p ≤ 0.001 (n = 5342). The ‘Not Significant’ category with p > 0.05 remains the same in Maps (**a**) and (**b**). Details of the statistics are supplied in “Methods” and the repository as noted. The results are based on the monthly data from June 2018 to May 2019. The maps are created by Geoda 1.18 (http://geodacenter.github.io/download.html).

Although a small portion of miners are hobbyists or believers, the majority of miners nowadays are mining for economic purposes. Undoubtedly, they should tend to concentrate in locations with a competitive advantage for mining. Our results demonstrate this tendency by aggregating and counting all hash rates of individual locations within each grid (Fig. 2). Eighteen top-tier grids (share of hash rate ≥ 1%) accounted for 61.8% of the total computing power during our study period. In fact, miners not only concentrate in a few grids but also cluster with each other in adjacent grids. Moran's *I* statistic is used to measure spatial concentration of mining activity (“Methods”). We find that the result suggests a strong rejection of the null hypothesis of spatial randomness (*I* = 0.65, *pseudo p* = 0.001 for 999 permutations, *z* = 97.8). In other words, mining activity demonstrated a strong tendency of concentration, in terms of computing power. We dig it further with Getis and Ord’s *G*_*i*_ statistic (“Methods”) to identify the hot spots (High-High cluster cores) of mining activity under different significance (Fig. 3). Our data extended from June 2018 to May 2019. The maps for spatial concentration and hot spots may change afterwards, which will be addressed in section “Spatial fluctuation”. In addition, mining activity is virtually concentrated in the format of mining pools. An increasing number of miners are now joining pools to optimize the scanning of hash values and share returns based on their computing power contribution^3,16^. In this analysis, we focus on the spatial phenomena in the physical world, so we will not pursue that in detail here.

Moran’s *I* statistic

3 $$ I = \frac{n}{{\mathop \sum \nolimits_{i = 1}^{n} \mathop \sum \nolimits_{j = 1}^{n} w_{ij} }}\frac{{\mathop \sum \nolimits_{i = 1}^{n} \mathop \sum \nolimits_{j = 1}^{n} w_{ij} \left( {X_{i} - \overline{X}} \right)\left( {X_{j} - \overline{X}} \right)}}{{\mathop \sum \nolimits_{i = 1}^{n} (X_{i} - \overline{X})^{2} }} $$

where *X*_*i*_ and *X*_*j*_ are the hash rates for grids *i* and *j*, $\overline{X}$ is the arithmetic mean of the hash rate for all grids, *w*_*ij*_ is the spatial weight between grids *i* and *j*, and *n* is equal to the total number of grids.

Getis and Ord’s *G*_*i*_ statistic

4 $$ G_{i} = \frac{{\mathop \sum \nolimits_{i = 1}^{n} \mathop \sum \nolimits_{j = 1}^{n} w_{ij} X_{i} X_{j} }}{{\mathop \sum \nolimits_{i = 1}^{n} \mathop \sum \nolimits_{j = 1}^{n} X_{i} X_{j} }},\quad \forall { }j \ne i $$

where *X*_*i*_ and *X*_*j*_ are the hash rates for grids *i* and *j*, *w*_*ij*_ is the spatial weight between grids *i* and *j*, and *n* is equal to the total number of grids.

### Spatial association

**Figure 4:**
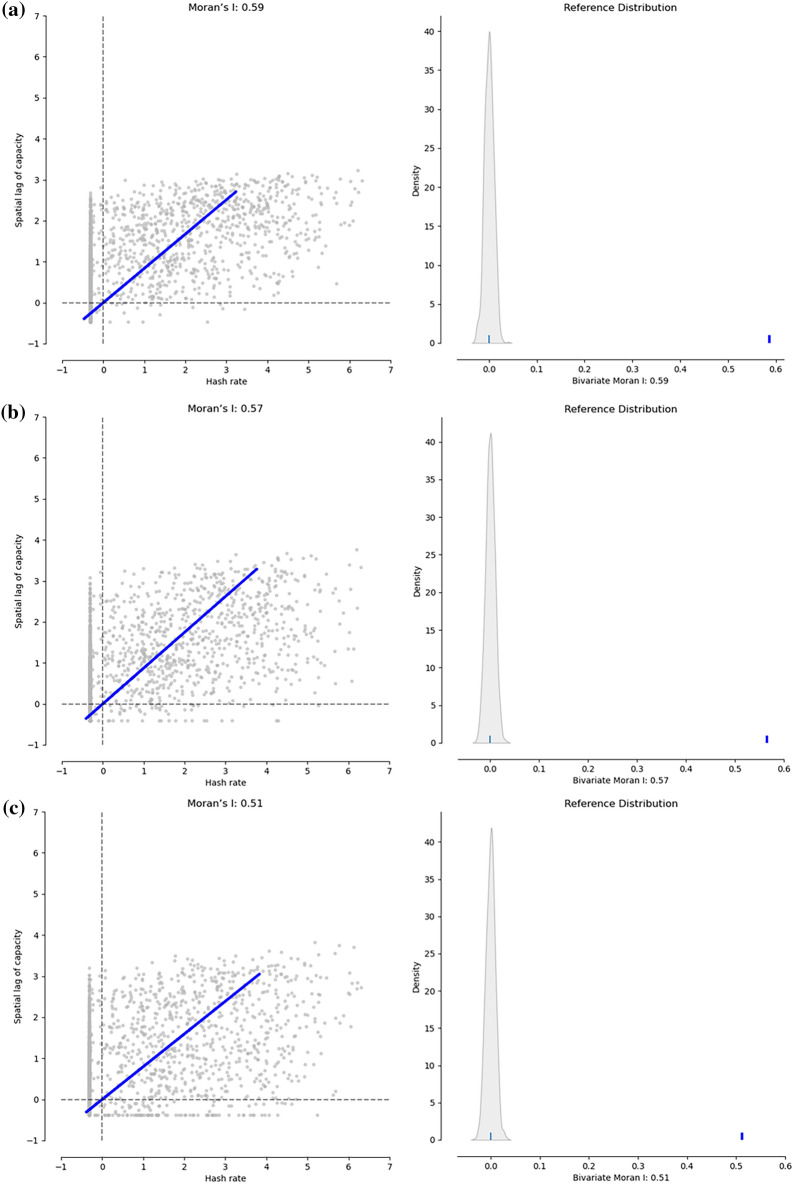
Bivariate Moran’s scatter plots and reference distributions between hash rate and different energy variables. (**a**–**c**) Bivariate Moran’s statistical results between the hash rate and capacity of all types of energy (**a**), fossil energy (**b**), and renewable energy (**c**) demonstrate the degree of spatial association between them. The scatter plot is depicted with the spatially lagged energy capacity on the y-axis and the original hash rate on the x-axis. The slope of the linear fit to the scatter plot equals Moran’s I. The reference distribution demonstrates the result by randomly permuting the observed values over the locations, which is depicted as a distribution curve in the left. The short line shows the value of Moran’s I, well to the right of the reference distribution. Details of the statistics are supplied in “Methods” and the repository as noted.

**Figure 5:**
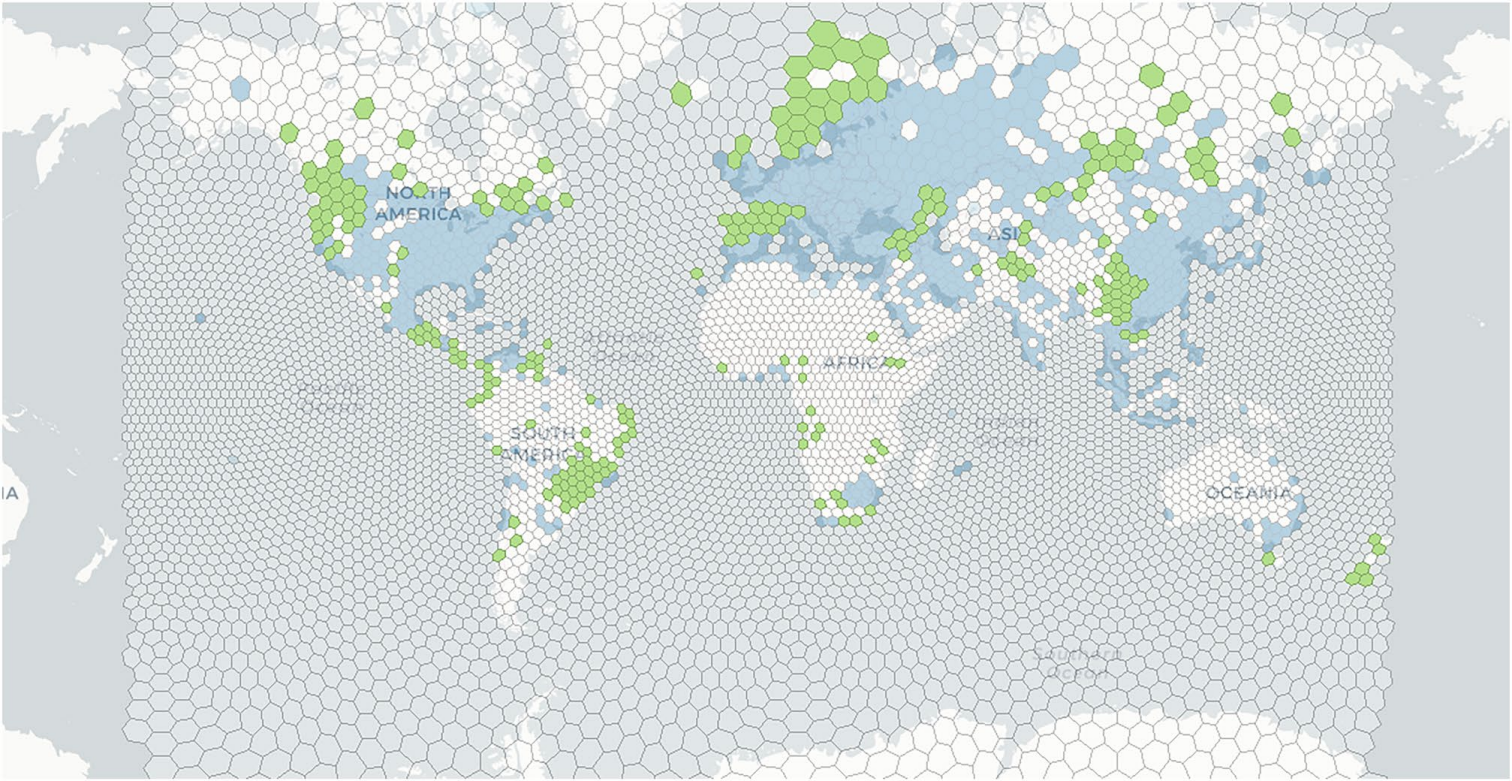
‘Spatial hit’ index indicates the potential locations suitable for renewable mining. Grids with ‘spatial hit’ index = 2 (i.e. suitable for renewable mining) are highlighted in green (n = 247). Details of the definition and calculation of the index are provided in “Methods”. The results associated with this map are shown in Supplementary Table S4. The results are based on the monthly data from June 2018 to May 2019. The map is created by Geoda 1.18 (http://geodacenter.github.io/download.html).

As illustrated in Eqs. (1) and (2) and corroborated by our interviews and other studies^7,11,15,16^, the most significant variable cost for mining activity is the electricity cost, which is used to power mining facilities. In this way, most miners should be inclined to locations that can provide cheap and constant sources of power. We put the global power plant data^23^ into the aforementioned hexagonal grid system and explored the bivariate Moran’s *I*_*xy*_ statistics (“Methods”) between hash rate and all energy types, fossil, renewable respectively. The results indicate a high significance of the spatial association between hash rate and all three energy variables (Fig. 4), though Moran’s *I* between hash rate and fossil energy (*I*_*hf*_ = 0.57) is slightly higher than that between hash rate and renewable energy (*I*_*hr*_ = 0.51). Furthermore, we designed a ‘Spatial-hit’ index (“Methods”) to identify areas suitable for renewable mining (Fig. 5), such as the Nordic (Hydro/Geothermal), US-Canada border areas (Hydro), US central (Wind), the Mekong River area (Hydro), and the Caucasus (Hydro).

Bivariate Moran’s *I*_*xy*_ statistic

5 $$ I_{xy} = \frac{n}{{\mathop \sum \nolimits_{i = 1}^{n} \mathop \sum \nolimits_{j = 1}^{n} w_{ij} }}\frac{{\mathop \sum \nolimits_{i = 1}^{n} \mathop \sum \nolimits_{j = 1}^{n} w_{ij} (X_{i} - \overline{X})(Y_{j} - \overline{Y})}}{{\mathop \sum \nolimits_{i = 1}^{n} \mathop \sum \nolimits_{j = 1}^{n} (X_{i} - \overline{X})(Y_{j} - \overline{Y})}} $$

where *X*_*i*_ and *Y*_*j*_ are the hash rate for grid *i* and the power capacity for grid *j*, $\overline{X}$ and $\overline{Y}$ are the arithmetic mean of the hash rate and the power capacity for all grids, respectively, *w*_*ij*_ is the spatial weight between grids *i* and *j*, and *n* is equal to the total number of grids.

It is worth noting that it is an adaptive process that mining activity demonstrates a strong spatial association with renewable energy. Renewable energy is not always the cheapest power source and sometimes might be expensive when transmission costs are also included. However, most types of renewable energy (e.g., hydro) bear some kind of ‘perishable’ characteristics, similar to those of fruits (cheap in original place and value down to zero if rotted). Renewable energy providers are willing to offer miners with heavy discounts during peak seasons^18^. Therefore, it becomes a perfect match between the surplus of renewable energy and the ‘portable’ mining activity. Miners did not realize this at the early stage, while they learned and reacted through continuous testing and iteration. This will be further addressed in the next section.

### Spatial fluctuation

**Figure 6:**
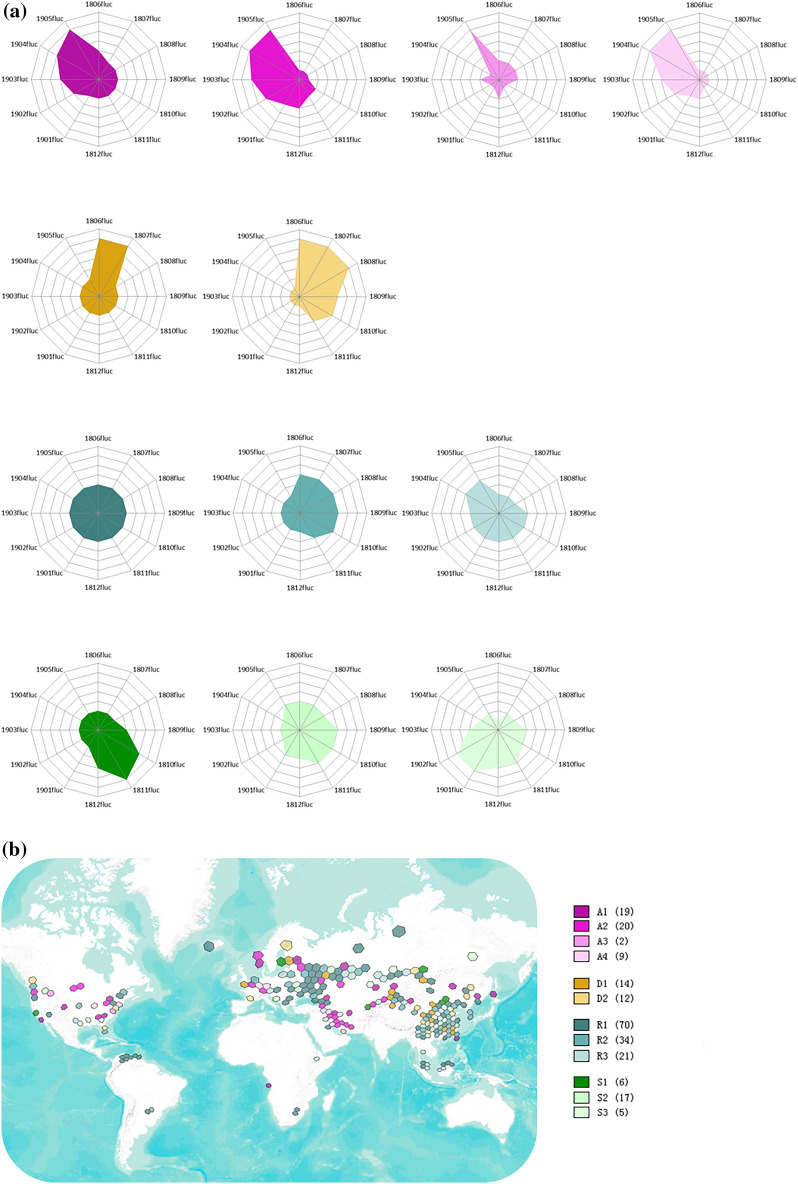
Classification of the grids with differentiated fluctuation patterns. (**a**) Grids with hash rate over 1500 TH/s (n = 229) are divided into twelve clusters in four groups. The twelve-month fluctuation indices of medoids are plotted in the radar chart as representatives of each cluster. (**b**) All the observed grids are plotted in Map (**b**) with their respective categories, sharing the sample colour scheme for each category in panel (**a)**. Details of the results are provided in Supplementary Tables S5, S6 and the repository. The results are based on the monthly data from June 2018 to May 2019. The map is created by Geoda 1.18 (http://geodacenter.github.io/download.html).

When we drilled down to monthly data, we found that mining activity fluctuated in space based on the rolling twelve-month hash rate from June 2018 to May 2019. Here we use 1500 TH/s as the threshold to select grids with at least 100 mining rigs for our analysis (Supplementary Note 4). In terms of the characteristics of monthly fluctuation, grids with hash rate over 1500 TH/s (n = 229) were observed and put into twelve clusters through cluster analysis with K-medoids (“Methods”). We further categorized twelve clusters into four groups with reference to the real operational environment: ascending, descending, relatively stable and seasonal fluctuation (Fig. 6).

Every fluctuating grid fluctuated in its own way, which might follow a combination of multiple patterns and can only be explicitly explained case by case. However, four primary patterns are studied and summarized here. (i) Price effect: the drop in the Bitcoin price drives mining profitability down, as illustrated in Eqs. (1) and (2). Large mining farms choose to migrate to locations with more cost advantages or update their mining machines, while most individual or small miners are reluctant to take immediate actions and wait for the suitable time to reopen their mining rigs. All these factors lead to a change in computing power in grids but to different degrees. (ii) Seasonal effect: some miners are accustomed to transfer periodically to leverage the discounts offered by suppliers within certain grids where there is surplus energy during the peak season (e.g., rainy season for hydropower grids). It also happens when these miners move back to their original locations during the off-season. (iii) Regulatory effect: attitudes from regulators dramatically influence the behaviours of miners in related grids. Favourable measures (e.g., subsidies, tax benefits) encourage miners to move in, while adverse measures (e.g., bans, carbon taxation) drive miners out. (iv) Iterative effect: initial mining activity may start randomly from the grids where early believers, tech geeks or speculators inhabit. Miners (in particular large ones) continue to learn and search for better mining locations. The process is iterative for optimal solutions, and the radius of search is expanded to adjacent grids and then gradually to the global scale. Thus, a considerable portion of computing power at the original grids is relocated to the well optimized grids. Unfortunately, only part of this pattern can be observed within our study since the anonymity of the Bitcoin network makes it nearly impossible to recognize early mining locations.

Spatial fluctuation is never ending. We notice that the recent change in regulatory policy towards Bitcoin mining in some jurisdictions (e.g., China’s crackdown in 2021) has intrigued a new round of spatial fluctuation and migration. Bitcoin mining activity is in the process of moving to achieve new spatial equilibrium^31,32^. We believe that the spatial analysis here will still be applicable in new circumstances.

## Discussion

We have presented an in-depth analysis of the spatial distribution and characteristics of Bitcoin mining through bottom-up tracking and geospatial statistics. Our research reveals that mining activity has been widely distributed all over the world. Meanwhile, the Moran's *I* statistics indicate a strong tendency of its spatial concentration and association with energy production locations. We also consider that the spatial distribution of Bitcoin mining fluctuates with diverse patterns, according to economic and regulatory changes. Moreover, as elaborated below, we believe that our findings from the spatial perspective will be beneficial to the related studies of blockchain technology, financial econometrics, sustainability and other fields.

First, our results show that in terms of presence, mining activity is widely distributed, which is in accord with the decentralized nature of blockchain technology. However, in terms of computing power, it has demonstrated a strong tendency of spatial concentration, particularly towards the locations with abundant and cheap energy supply. This phenomenon will increase the potential risk of a 51% attack^11^ and make the whole network more vulnerable due to regulatory changes, disasters or other constraints at specific locations. By pointing out this pitfall, we hope that our analysis could help to inspire the improvement of blockchain design which shall further assess the concentration risk not only from ownership structure but also from geographical distribution.

Second, we find that our analysis may help to gauge market valuation on cryptocurrency assets and contribute as an input layer for neural network pricing models. The number of Bitcoin participants, the amount of computing power and the diversity of mining locations shall be viewed as the indicators of how widely the consensus is accepted. These factors can be used to measure the positive feedback loop between network effects and security: broader consensus and more participants lead to higher value, which in turn attracts more miners and increases the security of transaction validation^26^. Moreover, the shift of mining capacity between Bitcoin and Altcoins shall indicate the price spillover effect among cryptocurrency assets and markets^10^. In fact, all of these can be quantified and structured as a separate input layer for neural network pricing models^33^ and be further assessed the viability by backtesting with historical data. If the effectiveness is proved, we believe that our methodologies and empirical findings may contribute to fixing price misalignment and producing novel insights on cryptoasset management.

Third, understanding spatial distribution of Bitcoin mining is crucial for making accurate estimations on sustainability issues and for providing practical suggestions on more eco-friendly mining activity. Spatial footprint of Bitcoin mining is one of the fundamental factors influencing the accuracy of estimations on energy consumption and carbon emissions^7,15^. Our results provide empirical evidence on the distribution and dynamics of mining activity, which help to estimate energy consumption and carbon emissions based on real situation instead of theoretical assumptions. Meanwhile, a series of studies have tried to give suggestions to reduce environmental impacts from cryptocurrency mining, such as less energy-intensive alternatives for the proof-of-work mechanism or guiding the computing power and energy to more meaningful usages^3,11,34^. These are all good attempts, though they might face technical challenges or still lack enough consensuses. Based on the spatial analysis, we suggest that a feasible way in the short term is to build a global framework to regulate mining activity and motivate miners to the locations of abundant renewable energy, particularly where with surplus capacity.

Last but not least, Bitcoin mining, which was originated from the mail exchange of cryptographic community, has brought substantial impacts on our real world in terms of market capitalization, spatial presence, energy consumption and carbon emissions. It also provides researchers with a remarkable experiment^35^ of the transparent rules, the general availability of data and the lively interaction. We hope that our analysis tackled from a spatial perspective will continue to inspire further cross-field communications among geography, sustainability, economics, finance and technology.

## Methods

### Data wrangling

The desensitized, geocoded and aggregated hash rate data (42,820 records in total) are given in Supplementary Table S1. The data were extracted from the million-level mining records of partnered pools. All sensitive or confidential data were desensitized to fully protect the privacy of all mining participants. Please note that the research team has no direct access to the original Internet Protocol (IP) addresses or any other privacy data. The IP addresses of mining activity were converted and geocoded into physical locations under the instructions given by the research team. IP addresses can be localized with ip2location.com or similar tools. The naming and classification of countries and regions are under the World Bank guidelines^36^. The hash rate data in Supplementary Table S1 are finally organized by month, geographical name, location identifier (with unique longitude and latitude coordinates) and hash rate (max, min and average value of each month). By ticking the location identifier, we detect Bitcoin mining activity in 6062 unique locations from 139 countries and regions (Supplementary Tables S2, S3) and visualize the results in Fig. 1.

We use the global power plant data^23^ (World Resources Institute, 2019) to indicate the abundancy of different types of energy in specific locations. We selected the following fields for analysis: geographical name, location identifier (longitude and latitude coordinates, gppd_idnr), primary_fuel and capacity_mw. Based on the commonly used energy types for mining activity, we categorize oil, gas, petoke and coal as fossil energy, hydro, geothermal, solar and wind as renewable energy and the remaining energy types as others.

### Gridding method and preprocessing

In this subsection, we explain what kind of geogrid system is used for analysis and how we connect the hash rate and power data to the grid system. A discrete global grid system (DGGS), looking like mosaics that cover the entire Earth's surface, is often used as the geometric basis for the building of geospatial data structures^37,38^. Our analysis builds on two publicly available hexagonal grid tools and resources^39,40^. The blank grid datasets (with no attribute data) are provided at the GitHub repository. The original hash rate, power data and grid information are stored in different layers. We then explore spatial join by counting and aggregating the hash rate and power data of each location into the associated grid (i.e. the point location of longitude and latitude is within the given grid). Thus, the point-based hash rate and power data are merged into the blank grid datasets. The results are provided at the repository and the script for the cross-layer operation is shared on GitHub. This step can also be achieved in a common geographic information system (GIS) environment.

In addition, we preprocessed some of the merged grid data as follows. (i) ‘HrateAvg’ is calculated as the twelve-month average of hash rate for each grid; (ii) ‘HrateShare’ is calculated as the percentage of each grid’s hash rate to the total; and (iii) both the hash rate and power capacity data of each grid are standardized as below.

6 $$ X\_ZS = ZSCORE(log_{10} (X + 1)) $$

where *X_ZS* is the standardized value by the Z-score method (*Z* = $\frac{x - \mu }{\sigma }$, $\mu$ is the mean of the sample, $\sigma$ is the standard deviation of the sample), and *X* is the raw value of the hash rate or power capacity.

### Spatial statistics

Two types of spatial weight matrices assisting further analysis are given in the repository. We calculate the spatial weights based on the Queen contiguity (sharing a common edge or vertex), which express the neighbour structure between the grids. The difference between two spatial weight matrices is whether the in-situ relation (i.e. the diagonal of the weight matrix) is considered.

Moran’s *I* statistic^41^ is the commonly used indicator of spatial concentration (autocorrelation) and illustrated in Eq. (3). In essence, Moran’s *I* statistic demonstrates the relation between a variable (in deviation from the mean) and its spatial lag (weighted average of adjacent grids). We use Python’s PySAL^42^ package to calculate Moran’s *I* statistic for the hash rate and the related reference distribution under 999 permutations. The results (*I* = 0.65, *pseudo p* = 0.001, *z* = 97.8) suggest a strong rejection of the null hypothesis of spatial randomness. Similarly, the bivariate Moran’s *I*_*xy*_ statistics between the hash rate and the capacity of all energy types, fossil and renewable are explored. The difference is that the bivariate statistic demonstrates the relation between one variable (hash rate) and the spatial lag of another variable (power capacity). In our case, the spatial lag of power capacity takes the in-situ value into consideration by applying the spatial weight matrix including diagonal. The results (*I*_*ha*_ = 0.59, *pseudo p* = 0.001, *z* = 60.7; *I*_*hf*_ = 0.57, *pseudo p* = 0.001, *z* = 59.6; *I*_*hr*_ = 0.51, *pseudo p* = 0.001, *z* = 53.6) indicate a high significance of the spatial association between hash rate and all three energy variables. The code, the settings for reproducibility and the outputs are archived on GitHub.

Getis and Ord’s *G*_*i*_ statistic^43^ is further used to indicate the location of the clusters of mining activity and illustrated in Eq. (4). The G^*^_i_ statistic is fine-tuned by including the in-situ value at the given grid (i.e., the spatial weight matrix including diagonal is applied). The G^*^_i_ statistic for each grid with an assessment of significance is calculated and compared with the global mean. A value larger than the mean suggests a hot spot (High-High cluster), while a value smaller than the mean indicates a cold spot (Low-Low cluster)^44^. The calculations are implemented and the results are visualized in Geoda^44^ (permutations = 999 and random seed = 123,456,789 as default for reproducibility).

We then introduce the concept of the ‘spatial hit’ index to determine the grids suitable for renewable mining, which is defined as

7 $$ Spatial\;hit\;index \, = \left\{ {\begin{array}{*{20}l} {2,\quad {\text{hash}}\;{\text{rate}} > 0 \cup {\text{capacity}}\_{\text{sl }}\;{\text{of }}\;{\text{renewable }} > {\text{fossil }}} \\ {1,\quad {\text{hash}}\;{\text{ rate}} > 0 \cup {\text{capacity}}\_{\text{sl }}\;{\text{of}}\;{\text{ renewable }} \le {\text{fossil}}} \\ {0,\quad {\text{hash }}\;{\text{rate}}\;{\text{ in}}\;{\text{ the}}\;{\text{ given}}\;{\text{ grid}} = 0} \\ \end{array} } \right. $$

The ‘spatial hit’ index is calculated to identify the grids where the value of the hash rate is greater than 0 and the spatial lagged capacity of renewable energy is greater than that of fossil energy. We can further use the original power data to trace back to the specific type of renewable energy (Hydro, Geothermal, Solar or Wind) for the grids with the ‘Spatial hit’ index equal to 2. The results (with the grids suitable for renewable mining highlighted in green) are also visualized in Geoda (Fig. 5).

### Cluster analysis

We use 1500 TH/s as the threshold to select the grids for cluster analysis of spatial fluctuation since it is difficult to predict the mining behaviour in grids with only a few rigs. During the period of our study, the hash rate for a typical mining rig was approximately 15 TH/s. A grid with hash rate over 1500 TH/s means that there are at least 100 mining rigs (equivalent to a small-sized mining farm) in the given grid. In this way, 229 grids out of all 933 grids with mining footprints are selected.

The fluctuation index measures the amplitude of the monthly fluctuation of hash rate in the given grid, defined as

8 $$ Hrate\_fluc = \frac{{M_{i} - \overline{M}}}{{\overline{M}}} $$

where *M*_*i*_ is the hash rate of each month (from June 2018 to May 2019) for the given grid and $\overline{M}$ is the twelve-month average of hash rate in that grid.

The results are stored in the grid datasets and shared on GitHub. The fluctuation index is further categorized as

9 $$ Hrate\_fluc\_code = \left\{ {\begin{array}{*{20}l} {6{ }\;\left( {{\text{skyrocketing }}\;{\text{increase}}} \right),\quad {\text{Hrate}}\_{\text{fluc}} > 1} \\ {5{ }\;\left( {{\text{significant }}\;{\text{increase}}} \right),\quad 1 \ge {\text{Hrate}}\_{\text{fluc}} > 0.75} \\ {{ }4{ }\;\left( {{\text{moderate}}\;{\text{ increase}}} \right),\quad 0.75 \ge {\text{Hrate}}\_{\text{fluc}} > 0.25} \\ {3\;{ }\left( {{\text{relatively }}\;{\text{stable}}} \right),\quad 0.25 \ge {\text{Hrate}}\_{\text{fluc}} \ge - 0.25} \\ {2{ }\;\left( {{\text{moderate }}\;{\text{decrease}}} \right),\quad - 0.75 \ge {\text{Hrate}}_{{{\text{fluc}}}} > - 0.25} \\ {1 \;\left( {significant \;decrease} \right),\quad - 1 \ge {\text{Hrate}}_{{{\text{fluc}}}} > - 0.75} \\ \end{array} } \right. $$

We use a classical partitioning technique of clustering (k-medoids with PAM) and perform the analysis through Python’s scikit-learn and scikit-learn-extra packages^45^. The code and the settings for clustering are provided in the repository. The clustering results for each grid (Supplementary Table S5) are appended into the grid datasets and visualized in Geoda. Twelve medoids (Supplementary Table S6), as representatives for each cluster, are extracted and further categorized into four groups based on the understanding of real mining operations: ascending, descending, relatively stable and seasonal fluctuations (Fig. 6).

### Robustness analysis

For spatial statistics, we use a computational approach based on permutation, which calculates a reference distribution for the statistic by randomly permuting the observed values over the locations. The statistic is computed for each of these randomly reshuffled data sets, which yields a reference distribution. This approach is not sensitive to potential violations of underlying assumptions, and is thus more robust^44^. It can be easily verified by replicating the process based on the code we provided on Github (https://github.com/jricb/bitcoin_mining_spatial_analysis).

For cluster analysis, the k-medoids method is used to minimize the sum of the distances from the observations in each cluster to a representative center (medoid) for that cluster^44^. In contrast to k-means and k-medians, k-medoids uses actual observations and will not move the center of the cluster towards the outlier^45^. Thus it can be more robust to noise and outliers. For instance, we test different methods and find that Grid 444 and 673 are appropriately divided into category 10 under k-medoids.

There are several algorithms to compute k-medoids. The approach we used is the partitioning around medoids (PAM) algorithm, which consists of two steps: Build (greedy initialization of the medoids from the dataset) and Swap (compute the cost of swapping one medoid with any non-medoid point and stop when there is no change in the objective function)^45^. We have observed that very similar results are yielded under different iterations and initialization settings. This can be also repeated by following the code and instructions we provided on Github.

### Study limitations

We acknowledge that there are some limitations in our analysis. First, the sample of mining records may not be fully representative. The pools we partnered with are BTC.com (primary) and AntPool (cross-check). Both were the subsidiaries of Bitmain (the former sold to BIT in 2021) and accounted for approximately 30% of the total hash rate during the period of our study. Large and medium-sized miners could allocate their computing power proportionally to the major pools, while small or individual miners might choose the pools nearby or perform solo mining. Thus, the sample may better represent large and medium miners instead of small miners. This implies that there might be more mining locations than detected and mining activity might distribute more evenly than demonstrated. Nevertheless, to the best of our knowledge, the dataset in this study should be the most comprehensive sample for mining activity at this level of detail.

Second, fake IP addresses may hinder the tracking of real locations. Some miners use virtual private networks (VPNs) to intentionally obfuscate their locations^15,17^ or manage their mining rigs through cloud services. However, it is reasonable to assume that the vast majority of miners have little incentive to use VPN given the resulting latency^15^. The connections from cloud services have been redirected to their original addresses as much as possible by the engineers of the partner pools. However, we cannot guarantee that the fake IP influence associated with the bottom-up approach has been completely eliminated.

Third, the approximation of renewable energy in each grid might be underestimated due to the data availability of global power plants. As noted by Byers et al.^26^, it is difficult to collect information on relatively new and small renewable power plants since they are not always recorded in public documentation. When we aggregate the power plant data from each location to approximate the abundancy of energy in the associated grid, this will lead to the fact that renewable energy is less extensively covered as fossil energy.

Finally, the spatial distribution and characteristics of mining activity are not stationary and continue evolving under different regulatory and economic scenarios. For instance, mining activity has shifted dramatically in space since a series of measures again Bitcoin mining was taken by the Chinese authorities in 2021. The latest data on mining actions have not yet been available. However, we are confident that our analytical methods and most of the results will still be applicable in new circumstances. Further studies are needed to observe the spatial dynamics of mining activity and draw conclusions about the ultimate form of similar self-driven production activity.

## Supplementary Information

Supplementary Information.

Supplementary Table S1.

Supplementary Table S2.

Supplementary Table S3.

Supplementary Table S4.

Supplementary Table S5.

Supplementary Table S6.

## Data Availability

All data used in the study are provided on GitHub (https://github.com/jricb/bitcoin_mining_spatial_analysis), Supplementary Information or publicly available online as noted.

## Code availability

Python Spatial Analysis Library (PySAL) can be downloaded from https://pysal.org/. Geoda can be downloaded from http://geodacenter.github.io/. Python’s scikit-learn and scikit-learn-extra packages can be downloaded from https://scikit-learn.org/ and https://scikit-learn-extra.readthedocs.io/, respectively. The code for customized spatial analysis is available on GitHub at https://github.com/jricb/bitcoin_mining_spatial_analysis.
