# Supplementary material for: Spatial analysis of global Bitcoin mining
Source: Sci Rep. 2022 Jun 23;12:10694. doi: 10.1038/s41598-022-14987-0 (PMC9226069; doi:10.1038/s41598-022-14987-0)
Supplement: Supplementary file 1 — Supplementary Information. [file 41598_2022_14987_MOESM1_ESM.pdf]

Supplementary Information for
Spatial analysis of global Bitcoin mining

Wei Sun[1,2], Haitao Jin[3], Fengjun Jin[1,2], Lingming Kong[4], Yihao Peng[4], Zhengjun Dai[3]

[1]Key Laboratory of Regional Sustainable Development Modeling, Institute of Geographic Sciences and Natural Resources Research, Chinese Academy of Sciences, Beijing, China
[2]College of Resources and Environment, University of Chinese Academy of Sciences, Beijing, China
[3]School of Computer, Beijing Information Science and Technology University, Beijing, China
[4]BTC Team, BIT Mining Limited, Hong Kong, China

Supplementary Note 1 | The term of 'Bitcoin' or 'bitcoin'

In most circumstances, people use Bitcoin, capitalized, to refer to the technology, the network or the cryptocurrency as a concept, and bitcoin, lowercase, for the unit of account. So we follow the convention here.

Supplementary Note 2 | The autonomous economy

The concept of the autonomous economy is originated from the observation of 'the operation systems which may have overall human supervision, but their moment-to-moment actions are automatic, with no central controller, such as logistical systems, electricity grid systems, future driverless-traffic systems'. They are 'self-organizing, self-configuring, self-healing and self-correcting'. 'These autonomous systems, as miniature economies, are highly interconnected and highly interactive, in which the agents are software elements 'in conversation with' and constantly reacting to the actions of other software elements'[30].

It is a concept evolving with our understanding on the digital/virtual economy. W. Brian Arthur is one of the most important contributors to this concept, with his publications in Nature Reviews Physics (https://www.nature.com/articles/s42254-020-00273-3 2021) Harvard Business Review (https://hbr.org/podcast/2019/06/the-autonomous-economy 2019), McKinsey Quarterly (https://www.mckinsey.com/business-functions/mckinsey-analytics/our-insights/Where-is-technology-taking-the-economy 2017).

Supplementary Note 3 | The surface area of Earth

According to the information from Wikipedia (https://en.wikipedia.org/wiki/Earth), we assume that 29.2% of Earth's surface is land.

Supplementary Note 4 | The threshold to select the grids for observation

During the period of our investigation, the hash rate for a typical mining rig (e.g. Antminer S9) is approximately 15 TH/s. Here we use 1,500 TH/s as the threshold to select the grids with at least 100 typical mining rigs, which is equivalent to a small-sized mining farm.

Supplementary Figure S1. Univariate Moran's scatter plot and reference distribution of hash rate. The Moran scatter plot below is depicted with the spatially lagged hash rate on the y-axis and the original hash rate on the x-axis. The slope of the linear fit to the scatter plot equals univariate Moran's *I* for hash rate. The reference distribution demonstrates the result by randomly permuting the observed values over the locations, which is depicted as a distribution curve in the left. The short line shows the value of Moran's *I*, placed at 0.65, well to the right of the reference distribution.

a

b

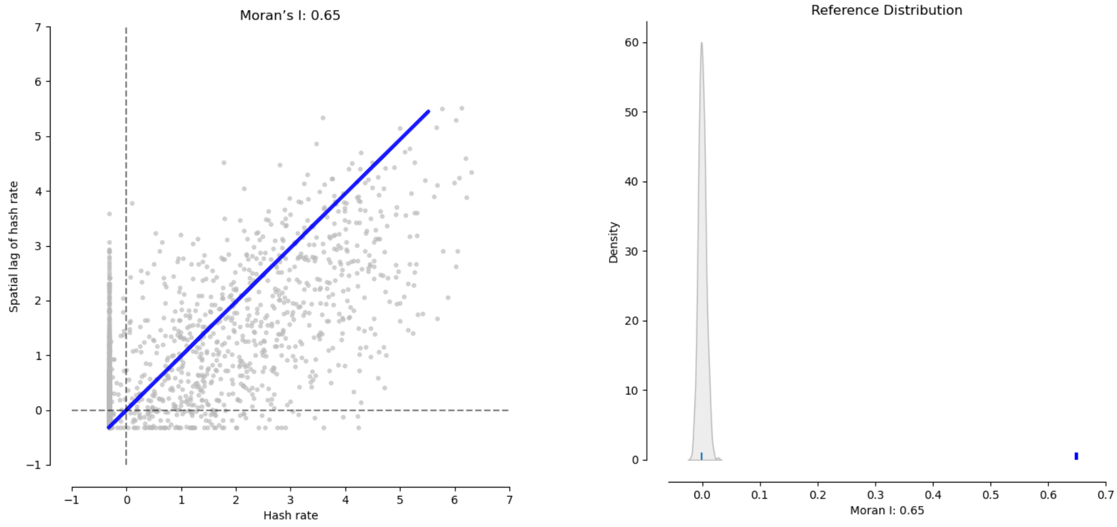

Supplementary Table S1. Aggregated hash rate by unique address. The Supplementary Table S1 includes all the desensitized, geocoded and aggregated hash rate data (42,820 records in total), which is fundamental for further analysis. The data are organized by month, geographical name, location identifier (with unique longitude and latitude coordinates) and hash rate (max, min and average value of each month). The data extended from June 2018 to May 2019.

The detailed information is provided in the attached spreadsheet 'Supplementary Table S1.xlsx'.

Supplementary Table S2. All unique locations: mining locations with unique longitude and latitude coordinates. Here we show the list of all mining locations detected with unique longitude and latitude coordinates, which is associated with the map in Figure 1.

The detailed list is given in the attached spreadsheet 'Supplementary Table S2.xlsx'.

Supplementary Table S3. Countries and regions with mining footprint. Here we show the name list of all 139 countries and regions where mining activity was detected. It is realized simply by removing duplicates from Supplementary Table S2.

The processed name list is given in the attached spreadsheet 'Supplementary Table S3.xlsx'.

Supplementary Table S4. 'Spatial hit' index for each grid. The ‘spatial hit’ index is introduced to identify the grids suitable for renewable mining (Methods). The supplementary table S4 includes the calculated index value for each grid, which is associated with the map in Figure 5. The result is based on the data from June 2018 to May 2019.

The detailed information is provided in the attached spreadsheet ‘Supplementary Table S4.xlsx’.

Supplementary Table S5. Clustering results for spatial fluctuation. Here we show the result of cluster analysis on the fluctuation index for each grid, which is associated with the map (b) in Figure 6. The result is based on the data from June 2018 to May 2019.

The detailed information is provided in the attached spreadsheet ‘Supplementary Table S5.xlsx’.

Supplementary Table S6. Twelve coded medoids. Twelve medoids derived from cluster analysis are coded to demonstrate the degree of fluctuation (Methods) and are categorized into four groups: ascending (A), descending (D), relatively stable (R) and seasonal fluctuations (S). The table includes the coded value and the assigned sub-group of twelve medoids, which is associated with the radar chart (a) in Figure 6. The data extended from June 2018 to May 2019.

The detailed information is provided in the attached spreadsheet ‘Supplementary Table S6.xlsx’.
